# A relational identity approach to study the antecedents of family supportive supervision

**DOI:** 10.3389/fpsyg.2022.1026352

**Published:** 2022-12-06

**Authors:** Pablo I. Escribano

**Affiliations:** Escuela de Negocios, Universidad Adolfo Ibáñez, Santiago, Chile

**Keywords:** work/life balance, interpersonal/relational processes, identity/identification, FSSB, supervisory-relationships

## Abstract

This research focuses on the antecedents of family supportive supervisor behaviors (FSSB) – the support from supervisors that can help employees manage their competing demands across work and nonwork domains. Drawing on theories of relational identity and self-construal, I conceptualize subordinates’ likeability (interpersonal abilities) and competence (task abilities) as antecedents of family supportive supervisor behaviors, and examine whether supervisors’ relational identification with subordinates mediates this relationship. In addition, I also examine the extent to which this mediation depends on the level of relational self-construal of supervisors. Data from 205 subordinates and 84 supervisors from a Chilean private bank and results support the hypothesized mediated moderation model. While supervisors’ relational identification with subordinates fully mediates the relationship between competence and family supportive supervisor behaviors, supervisors’ relational identification with subordinates partially mediates the relationship between subordinates’ likeability and family supportive supervisor behaviors. Further, supervisors’ relational identification with subordinates mediates the relationship between likeability and family supportive supervisor behaviors when supervisors’ relational self-construal is high to medium but not when it is low. Overall, this research offers a novel lens for better understanding subordinates as more than mere recipients of supervisory behaviors.

## Introduction

Family supportive supervisor behaviors (FSSB) are actions taken by supervisors that help subordinates address and manage their work and personal life responsibilities (Li and Bagger, 2011). Supervisors can be consciously unsupportive by expecting allegiance to work at the expense of family, or they can be unconsciously unsupportive by being unaware of what they can do to support employees in the organizational context. Supervisors, on the other hand, can be supportive by taking an interest in their employees’ personal lives, resolving scheduling conflicts, providing flexibility, and evaluating results rather than “face time” (Hammer et al., 2007). The importance of such extra-role, discretionary supervisory behavior stems from the fact that it is linked to a number of desirable outcomes such as employee creativity (Zhou et al., 2022), task performance (Wang et al., 2013), employee perceived health (Rofcanin et al., 2020), and perceived promotability of the employee (Rofcanin et al., 2018), among others. Because of its practical importance for organizations and employees, scholars have devoted a large effort to better understand the antecedents of FSSBs. Generally, this work has focused on individual or situational determinants that affect the level of support received by subordinates. While the studied individual differences include supervisor’s time pressures in core-tasks, empathy, gender, childcare responsibilities, and hierarchical position (i.e., Li and Bagger, 2011; Epstein et al., 2015; Pan et al., 2021) the contextual features that have captured the attention of work-family scholars include the presence of an organizational culture that is supportive of family and the availability of formal family-supportive organizational policies and practices (i.e., Hammer et al., 2007; Epstein et al., 2015).

In addition to such individual and situational factors, recent theoretical advances have conceptualized FSSB as a dyadic phenomenon, stating that “supervisor-employee dyads are very important to the enactment of work-life support” (Kossek et al., 2018, 16). In fact, it has been theorized that supervisors engage in FSSB selectively, such that the extent to which a supervisor supports a specific subordinate depends on features of the particular dyadic relation between the supervisor and subordinate (Straub, 2012; Sargent et al., 2022). Accordingly, research has shown empirically that a significant portion of the variance in FSSB resides at the dyadic or relational level (e.g., Epstein et al., 2015). Existing studies have focused on demographic factors and shown that when supervisors in a cross-gender dyad had more child care responsibilities than their employees, the supervisors tended to provide more family support (Li and Bagger, 2011). Alternatively, with a focus on race and not on childcare responsibilities, Foley et al. (2006) found that supervisors provided more family support to subordinates who were similar in either gender or race, with the level of support being highest when subordinates were similar in both gender and race to supervisors. Although these initial findings contribute to our understanding on the antecedents of FSSB, to move forward it is important to understand FSSB as resulting from the history of interactions between supervisors and subordinates.

The purpose of this study is to increase our understanding of the antecedents of FSSB in supervisor-subordinate dyads by examining the role of supervisors’ relational identification with subordinates. Building on the notion that “[as] relational identification grows, one tends to monitor the costs of social exchanges less closely and to take pleasure in contributing to the welfare of the role-relationship because of the inclusion of the relationship in one’s own self-concept” (Sluss and Ashforth, 2007, 19), I propose that supervisor’s relational identification with each subordinate [i.e., internalizing the attributes of the other person that bear on the role relationship as a partial definition of who one is (Sluss and Ashforth, 2007)] explains the extent to which they engage in FSSB toward each subordinate. Further, on the basis of insights from relational-identity theory (e.g., Andersen and Chen, 2002; Sluss and Ashforth, 2007), I argue that likeability and competence perceptions act as antecedents of relational identification with subordinates. Therefore, these two dimensions of interpersonal perceptions that develop over time as people work together (Cuddy et al., 2011) represent an initial step in explaining why supervisors support subordinates to a greater or lesser extent.

Furthermore, I argue that a more complete study of the antecedents of relational identification as a state requires consideration of the relational self-concept of supervisors as a trait. As a trait, supervisors with strong relational self-concept regard close interpersonal relationships with subordinates as the primary basis for self-definition, and their self-worth is derived from the positive regard of others (Johnson et al., 2012). Incorporating this insight with relational identity theory, I propose that likeability and competence perceptions will have little or no effect on FSSB *via* relational identification when supervisors are low in relational self-concept. Therefore, gaining theoretical depth on the boundary conditions of the hypothesized mediated model.

In the present study I make several contributions to the literature. First, by taking the perspective of supervisors and using relational identity theory to investigate the role of supervisors’ relational identification in their behavioral response to their perceptions about subordinates’ likeability and competence, the present study helps better understand the nomological network of FSSB (Crain and Stevens, 2018). In particular, I add to the literature by conceptualizing FSSB as a by-product of relational identification with subordinates. Second, I contribute to the existing knowledge on relational identity by better understanding the antecedents of supervisors’ relational identification with subordinates. Although the relational identity lens has been used in the work-life literature to study supervisor-subordinate work relationships from the perspective of subordinates (i.e., Wang et al., 2013), this is the first study to look at supervisors’ discretion to engage in supportive behaviors toward subordinates. Along these lines, I contribute to the existing literature by conceptualizing likeability and competence as antecedents of relational identification with subordinates. Third, I investigate the boundary conditions that underpin the relationship between supervisors’ relational identification and their perceptions of the likeability and competence of their subordinates. Here I consider relational self-concept as a supervisory trait that determines when supervisors’ perceptions of subordinates’ likeability and competence will lead to FSSB through relational identification with subordinates.

## Theoretical background

The focus of this study is on the antecedents of family supportive supervisor behaviors (FSSB), understood as the behaviors exhibited by supervisors that are supportive of a subordinate’s family role (Hammer et al., 2009). Although the FSSB construct was defined as an informal type of social support that is family specific, the actual items from Hammer et al. (2009, 2013) measures refer more generally to the nonwork domain. Crain and Stevens (2018), in a review of the FSSB literature, note that although the FSSB scales do not directly refer to family, the more general nonwork items allow for varying interpretations of family. Supervisors may assist subordinates in accommodating demands, needs, and conflicts from whomever they personally consider to be family. Accordingly, supervisors may support a subordinate in their family role by helping the subordinate feel comfortable when talking about conflicts between work and non-work, by demonstrating effective behaviors in how to juggle work and non-work responsibilities, by working effectively with the subordinate to creatively solve conflicts between work and non-work, and by organizing the work assigned to the subordinate in ways that jointly benefit the department or unit and the subordinate.

Considering the behaviors included in the overall definition of FSSB, it is apparent that it fits within the general class of discretionary extra-role behaviors (Straub, 2012). Supervisors who engage in FSSB, like those who engage in other-oriented discretionary behavior, go above and beyond the call of duty to do something for the benefit of a subordinate (Jin et al., 2022). In light of the fact that supervisors are already under pressure to perform their core responsibilities, researchers have focused on gaining a deeper understanding of why supervisors would go out of their way and support subordinates on matters related to their personal life. To address this question from the perspective of supervisors, scholars have begun to investigate the factors that influence the extent to which they engage in FSSB. For example, previous research has shown that FSSB acts as a mediating mechanism between supervisors’ perceived organizational friendly culture in terms of time pressures and subordinates’ perceived overall health and satisfaction with work-family balance (Rofcanin et al., 2020). Moving the attention from the organizational norms and pressures that influence supervisors’ behaviors to the supervisors’ internal forces influencing their ability and willingness to engage in FSSB, Pan (2018) found a positive relationship between supervisor workaholism and FSSB, which was strengthened by supervisors’ perceptions of subordinate family-to-work conflict. Moreover, Pan et al. (2021) report that the positive relationship between a supervisor’s work-to-family conflict and their subordinates’ work-to-family conflict can be explained by the fact that supervisors tend to prioritize core tasks and exhibit less FSSB. Overall, the existing literature indicates that supervisors’ perceptions have a significant impact on their discretionary engagement in FSSB, which in turn influences how subordinates can manage work and non-work responsibilities.

However, in the current literature, the supervisor’s discretion in deciding whether to engage in FSSB with each subordinate is not addressed. Previous research has suggested that supervisors may engage in FSSB selectively (Straub, 2012; Sargent et al., 2022), but our current understanding of the factors that may explain why a supervisor is more willing to engage in FSSB with some subordinates than with others is rather limited. With regards to the purpose of better understanding the nomological network of FSSB, Ashforth et al. (2008) suggest that the behavioral choices an individual makes reflect the individual’s perception of the self: “The concept of identity helps capture the essence of who people are and, thus, why they do what they do – it is at the core of why people join organizations and why they voluntarily leave, why they approach their work the way they do and why they interact with others the way they do during that work” (Ashforth et al., 2008, 334). That is to say, the way individuals act is an outcome of the meaning that we derive from our identities. Hence, in this article, I propose that identity-related factors can explain the attitudinal aspects underlying the supervisor’s motivation to engage in FSSB. Following the identity line of thought and focusing on the work relationship between a supervisor and a subordinate, I argue that FSSB is the behavior that emerges as a supervisor identifies with a particular subordinate in a work-relationship.

### Relational identity theory

Sluss and Ashforth (2007), in their seminal piece, expanded the definition of identification to include interpersonal relationships, such as those between supervisors and direct reports, despite the fact that identification has traditionally been understood to refer to one’s sense of self in relation to a particular group, occupation, profession, or organization (Anglin et al., 2022). A relational identity enables an individual within a dyad to respond to the question “who are we?,” thereby establishing the perceived nature and significance of the role-relationship for that individual (Andersen and Chen, 2002). Relational identity, or the nature of one’s role-relationship, is made up of both role-based and person-based identities. Sluss and Ashforth (2007) assert that a role-based identity is made up of the goals, values, beliefs, norms, interaction styles, and time horizons that are usually associated with the role. A person-based identity is made up of the personal qualities of the role occupant that affect how the role-based identity is displayed in practice. This means that the person-based identity shapes how the role-based identity is enacted as relational partners interact (Sluss and Ashforth, 2007). From the perspective of a supervisor working at a bank, for example, the subordinate’s role-based identity may include duties like writing reports and attending to reviews, while the person-based identity may include ways in which duties are done, like demonstrating honesty and agreeableness. Relational identity is a perception of the individual in relation to the role-relationship, according to Sluss and Ashforth’s (2007) work on relational identity theory, so I focus on the supervisor’s perception of the relational identity with a subordinate.

Sluss and Ashforth (2007) make a distinction between relational identity and relational identification within their theory of the relational self. While a relational identity is the nature of the role relationship, relational identification is the “partial definition of oneself [the focal individual] in terms of [the] role relationship” (Sluss and Ashforth, 2007, 15). Identification is the process of integrating the significant other into one’s self-concept or identity because the other person is self-defining (one wants to be like or get the qualities of the other) and meets the person’s underlying needs for self-enhancement, self-expansion, and meaning (Ashforth et al., 2008). In other words, relational identification is the degree to which one includes the role relationship in one’s self-concept. It has been hypothesized that relational identification increases the likelihood of highly personalized relationships because people are more likely to direct their positive actions and affections toward those with whom they identify (Humberd and Rouse, 2016). In the context of this research, a supervisor has role-relationships with each of the subordinate in their work-unit and the relationship with each subordinate will engender its own degree of relational identification. Moreover, the extent to which each of those relationships become self-defining for the supervisor is expected to vary–the more attractive or desirable a particular relational identity (the relationship with a specific subordinate) is perceived to be, the higher the level of relational identification (Sluss and Ashforth, 2007).

Regarding the antecedents of relational identification, or what makes a role-relationships to be perceived as more or less desirable, scholars have theorized about the cognitive components of identity that may lead to identification (Sluss and Ashforth, 2007, 2008). Consistent with the person-based and role-based components of relational identity, perceived likeability and competence echo two universal dimensions in social cognition (Fiske et al., 2007), and forms of both have been shown to affect how supervisors evaluate subordinates (e.g., Borman et al., 1995; Wayne et al., 2022). The likeability (warmth) dimension captures traits related to the perceived intent for good or ill, including friendliness, tolerance, helpfulness, and sociability (Fiske et al., 2007). The competence dimension captures perceptions of general ability or expertise (Cuddy et al., 2011). For supervisors, these two dimensions independently contribute to the impression that a subordinate is a valuable target for building and sustaining a work relationship that is meaningful for the self–acting as antecedents of relational identification with subordinates. Previous research indicates that individuals perceived as warm or competent within work-units are perceived as more influential within the work-unit and tend to emerge as informal leaders (Fransen et al., 2018). Therefore, these two dimensions tend to influence supervisors in their evaluation of the relational identity, thereby predisposing them to feel affinity for a target subordinate. In this paper, I take the perspective of supervisors and use relational identity theory to investigate the role of supervisors’ relational identification in their behavioral response to their perceptions about subordinates’ likeability and competence. To this end, I first focus on the antecedents and consequences of relational identification to then hypothesize the mediation and moderation effects.

## Hypotheses

### Likeability and competence as antecedents of relational identification

Generally, subordinates are not passive role-takers and have some latitude in enacting their roles in ways that are consistent to their own needs, values, and preferences (McAllister et al., 2007) and supervisors are well aware of that (e.g., Dufour et al., 2021). As a result of their interactions over the course of their working relationship, a supervisor’s overall impression of a subordinate can be summed up by how likeable and competent the subordinate is in the eyes of the supervisor. Here, I contend that supervisors will tend to identify more with those subordinates who have good intentions (likeability dimension) and that can meet the norms and expectations associated with their work role (competence dimension) because those individuals are better candidates for building and sustaining satisfactory work relationships (Gross et al., 2021). Just as perceived competence and likeability perceptions have been related to cognitive and affective trust (i.e., Oo et al., 2022), I expect these two factors to work together to inform a supervisor that their subordinate is a good relational partner with whom to form and sustain a meaningful work relationship. Therefore, the level of relational identification with a target subordinate is likely to be predicted by the perceived likeability and competence supervisors associate with that subordinate.

Existing research in supervisor-subordinate dyads indicates that likeability and competence perceptions have a significant impact on the value supervisors place on each work relationship. Supervisors perceived likeable subordinates to be more similar (Wayne et al., 1997), demonstrating that likeability is a strong factor explaining the extent to which the supervisor’s definition of self includes elements of the subordinate. More competent followers are more likely to be better performers and more aligned with the supervisor’s role-based identity expectations, causing supervisors to have a higher regard for such subordinates (Scully et al., 1995). Indeed, previous research has shown that supervisors’ liking for subordinates, as well as their perceived competence, predicted the quality of the supervisor-subordinate work relationship (Liden et al., 1993) and the commitment of the subordinate (Leslie et al., 2012) as perceived by the supervisor. Supervisors’ perceptions of likeability and competence predict the value they place on a target subordinate’s contribution to goal achievement in comparison to their peers, where likeability and competence independently explain how desirable they are as subordinates (Diekmann et al., 2015). Together, these findings suggest that subordinates perceived as likeable and competent are more likely to meet supervisors’ task and psychological needs. As a result, likeability and competence perceptions should be positively related, independently, to supervisors’ relational identification with subordinates. Building on this logic, I hypothesize that:

*Hypothesis 1a*: The supervisor's likeability perception of the subordinate is positively related to the supervisor's relational identification with the subordinate.

*Hypothesis 1b*: The supervisor's competence perception of the subordinate is positively related to the supervisor's relational identification with the subordinate.

### Family supportive supervisor behaviors as a result of relational identification

I propose that relational identification with subordinates will positively relate with FSSB. Here, I conceptualize FSSB as a discretionary behavior that functions as the behavioral enactment of relational identification. As Ashforth et al. (2008) suggest, the behavioral choices individuals make reflect who the individual thinks he or she is. Identity explains why people do what they do. “The concept of identity helps capture the essence of who people are and, thus, why they do what they do–it is at the core of why people join organizations and why they voluntarily leave, why they approach their work the way they do and why they interact with others the way they do during that work.” (Ashforth et al., 2008, 334). In other words, identity serves as an important sense–making function that is manifested through behavior.

Theoretically, one would expect supervisors who have a high level of relational identification with a subordinate (as perceived and reported by the supervisor) to show more care and concern about issues related to the subordinate’s personal life (as perceived and reported by the subordinate) for a variety of reasons. First, FSSB is consistent to the relational identity lens since it is conceptualized as a discretionary behavior done by supervisors to benefit subordinates. Therefore, it is a behavior that is relational in nature (Hammer et al., 2011). Second, relational identification entails an expansion of the self to include those facets of complementary roles and its incumbent(s) that hold a role-relationship. Self-expansion is closely related to empathy, reflected by an incapacity to discriminate between oneself and one’s relational partner actions, thoughts, and feelings (Gardner et al., 2002). Thus, a high relational identification leads supervisors to personally feel the relational partner’s concerns, and by providing support, they are at least partially helping themselves too. In fact, it has been theorized that in order to protect the relationship and seek the welfare of the subordinate supervisors can engage in person-focused discretionary behaviors like listening, being available for emotional support, counseling, and demonstrating concern (Eberly et al., 2011); which overlaps with the type of behaviors that work-family scholars conceptualize as FSSB. Third, supervisors who have strong relational identification with their subordinates consider their subordinates to be psychologically close to them. When supervisors define themselves in terms of their close relationships with their subordinates, they are concerned with their subordinates’ welfare and needs (Cooper and Thatcher, 2010) and feelings of self-worth are derived from the well-being of subordinates’ (Brickson, 2000). Indeed, when supervisors feel psychologically close to a subordinate, supervisors are increasingly aware of their relational partner’s personal needs, and in an expressive and non-calculative manner direct a vast amount of effort to help subordinates achieve personal objectives (Yoon, 2017). Thus, relational identification toward a subordinate is expected to be positively related to FSSB.

From an empirical perspective, no previous research has examined specifically the relationship between supervisor relational identification and FSSB. However, some supporting evidence can be extrapolated from existing research. For example, in two studies using data reported by subordinates, previous scholars found that FSSB is positively related to relationship quality (Bagger and Li, 2014) and relational identification with supervisor (Wang et al., 2013). Similarly, using supervisory reports, it has been found that behaviors signifying identification (e.g., showing interpersonal concern, fulfilling expected obligations) strengthen the bonds between employees and their supervisors, measured as relationship quality (Graen and Uhl-Bien, 1995; Settoon et al., 1996). Indeed, Epstein et al. (2015) report that relationship quality with subordinates, as perceived by supervisors, is one of the strongest situational predictors of FSSB. Collectively, these findings suggest that from the supervisor’s perspective FSSB–as a behavioral manifestation–is a mechanism by which supervisors ensure the well-being and maintenance of the relationship. Therefore, I hypothesize:

*Hypothesis 2*: The supervisor's relational identification with the subordinate is positively related to the subordinate's perception of the supervisor's family supportive behaviors (FSSB).

### Relational identification as a mediator

Combined, Hypotheses 1 and 2 convey that likeability and competence perceptions are positively associated with FSSB *via* relational identification with subordinates. Building on relational identity theory, I argued that likeability (H1a) and competence (H1b) perceptions contribute independently to engender strong relational identification with their subordinates. Supervisors are close to their subordinates (i.e., high relational identification) when they perceive there to be an overlap in selves with their subordinates (see Aron et al., 2001). When supervisors define themselves in terms of their close relationships with their subordinates, they are concerned with their subordinates’ welfare and needs because of the inclusion of the relationship in their own self-concept (see Cooper and Thatcher, 2010). Such perceived oneness, captures by relational identification, is an important pathway through which empathic concerns turn into actual helping behaviors (Cialdini et al., 1997)–FSSB in this case (H2). Similarly, from an identity perspective lens (e.g., Ashforth et al., 2008), this pathway explains how perceptions lead to behaviors that are consistent with the affective state involved in the relationship with the target. Therefore, I hypothesize:

*Hypothesis 3a*: The supervisor's relational identification with the subordinate mediates the relationship between the supervisor's likeability perception of the subordinate and the subordinate's perception of the supervisor's family supportive behaviors (FSSB).

*Hypothesis 3b*: The supervisor's relational identification with the subordinate mediates the relationship between the supervisor's competence perception of the subordinate and the subordinate's perception of the supervisor's family supportive behaviors (FSSB).

### Relational self-concept as a moderator

Although relational identity scholars have conceptualized the relationship between relational identification and its antecedents as stable across individuals, self-concept scholars propose that this relationship depends on the level of relational self-concept (in this case, of supervisors). As a self-regulatory variable, the self-concept guides people toward certain work attitudes and behavior intentions. People with a highly relational self-concept think and act in ways that nurture close relationships (Cross and Morris, 2003), and they tend to think of themselves in terms of their relationships with close others (Cross et al., 2000). People with a highly relational self-concept see close dyadic relationships as the primary basis for self-definition, and their sense of self-worth comes from the positive regard of others.

Based on theories of self-concept, I argue that the relational self-concept moderates the positive relationship between likeability and competence perceptions and relational identification. More specifically, I hypothesize that the relationship is stronger for supervisors with higher relational self-concept. In studies in which relational self-concept is conceptualized and measured as a chronically accessible trait, individuals with a high relational self-concept were found to be more likely to detect, encode and process stimuli which trigger one’s ability to develop relationships with others. Cross et al. (2000) found that for individuals with a strong relational self-concept, the ability to form and affirm relationships is a source of positive affect and self-esteem. Individuals who score high on the relational self-concept scale are also able to predict people’s values and beliefs more accurately and are more optimistic about people in their first encounters than are individuals who score lower on the scale (Cross and Morris, 2003). Finally, within work settings, individuals with a strong relational self-concept tend to derive to a greater extent the affect felt toward others from their own history of dyadic interactions than individuals who a low level of relational self-concept (Johnson et al., 2006). Together, these results suggest that people with a strong relational self-concept are more likely to look for, remember, and use information about their past experiences with someone, and to use this information in a consistent way to judge how close they feel to that person. By extending this rationale to the context of supervisor-subordinate dyads, I hypothesize:

*Hypothesis 4a*: The supervisor’s relational self-concept moderates the positive relationship between the supervisor's likeability perception of the subordinate and supervisor's relational identification with the subordinate such that the relationship is stronger for supervisors with higher relational self-concept.

*Hypothesis 4b*: The supervisor’s relational self-concept moderates the positive relationship between the supervisor's competence perception of the subordinate and supervisor's relational identification with the subordinate such that the relationship is stronger for supervisors with higher relational self-concept.

**Figure 1:**
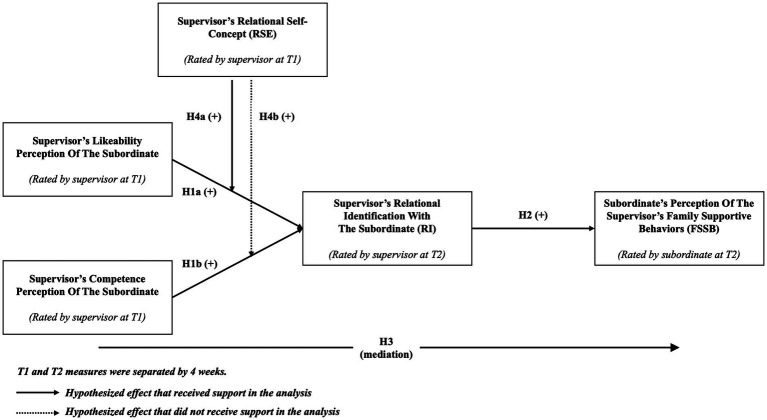
Hypothesized model.

Implicit to Hypotheses 4a and 4b is that relational self-concept also moderates the indirect effect of perceived likeability on FSSB *via* relational identification, and of perceived competence on FSSB *via* relational identification, respectively. Theoretically I propose that the higher the supervisor’s relational self-concept, the greater the impact of likeability and competence perceptions on FSSB. More specifically, as the supervisor’s relational self-concept grows, their perceptions of the subordinate will have a greater influence on their relational identity. As a result, the indirect effect (i.e., mediated effect) of the supervisors’ likeability and competence perceptions on the subordinate’s perception of FSSB will become stronger as the relational self-concept grows (see Figure 1).

## Materials and methods

### Participants and procedure

Data from full-time supervisors and subordinates working in a Chilean private bank were collected for this study. Before the study began, the company supervisors and subordinates were briefed by the HR manager about the purpose, procedure, and confidentiality of the study using the bank’s official communication channels. The link to access the survey was sent to their work emails (provided by HR), supervisors and subordinates could complete the survey during work hours, and although participation was voluntary, respondents were informed that in exchange of their time and effort I will donate 1.5 euros to a local charity for each participant completing the survey.

One hundred fifty-three supervisors (all at the same hierarchical level and not holding managerial responsibilities) and their subordinates (i.e., 522) were invited to participate in this study. To alleviate common-method and common-source concerns (Spector et al., 2019), I separated the data collection into two rounds over a period of 4 weeks. At Time 1, supervisors completed a survey that included a measure of their relational self-concept and measures for rating the likeability and competence of each subordinate under their direct supervision. At Time 2, 4 weeks later, supervisors completed a survey where they were asked to assess the relational identification with each subordinate under their direct supervision and subordinates completed a survey where they assessed the family supportive supervisor behaviors displayed by their supervisor toward themselves. Demographic information of supervisors and subordinates were retrieved from the company’s official HR data.

Building on classical test theory, Newman (2014) asserts that all available information should be used in the data analysis. In that context, a respondent is considered usable when for each of the constructs of interest, the respondent completed at least one of the items used to measure the construct (within construct missingness). From the 153 supervisors invited, 97 had usable responses for time 1 and 99 had usable responses for time 2. From the 522 subordinates invited to participate, 322 gave usable responses at time 2. After using email addresses as IDs to match survey responses, there were 225 paired responses (84 supervisors and 225 subordinates) and 205 responses had no missing values (84 supervisors and 205 subordinates). The 84 supervisors in this sample had an average age of 43.93 years, an average organizational tenure of 10.71 years, and 54% of all supervisors were female. The 205 subordinates had an average age of 39.16 years, 50% were married or cohabiting, an average organizational tenure of 7.63 years, and 38% of all subordinates were female. On average, the supervisors and subordinates had worked together for 3.26 years.

### Measures

Back-translation procedures were followed to translate the scales from English to Spanish and back. All variables were measured using multi-item, five-point Likert scales, where 1 indicates “strongly disagree” and 5 means “strongly agree”.

#### Perceived employee likeability

Perceived employee likeability was measured in time 1 using four items from Turnley and Bolino’s (2001) likeability scale. Supervisors were asked to rate each of their subordinates in terms of their level of agreement to the following statements: (1) “*This subordinate is a likable person at work*,” (2) “*This subordinate is a cooperative person at work*,” (3) “*This subordinate is a nice person at work*,” and (4) “*This subordinate is a pleasant person at work*.” I obtained a Cronbach’s alpha of 0.90 and averaged the items to form an index.

#### Perceived employee competence

Perceived employee competence was measured in time 1 using four items from Turnley and Bolino’s (2001) likeability scale. Supervisors were asked to rate each of their subordinates in terms of their level of agreement to the following statements: (1) “*This subordinate is a competent person at work*,” (2) “*This subordinate is an intelligent person at work*,” (3) “*This subordinate is a talented person at work*,” and (4) “*This subordinate is an accomplished person at work*.” I obtained a Cronbach’s alpha of 0.92 and averaged the items to form an index.

#### Relational self-concept

Relational self-concept was assessed with the Levels of Self-Concept Scale (LSCS: Johnson et al., 2006) in time 1. More specifically, relational self-concept was assessed by supervisors with the five-item Concern for Others sub-scale: (1) “*If a friend was having a personal problem, I would help him/her even if it meant sacrificing my time or money*,” (2) “*I value friends who are caring, empathic individuals*,” (3) “*It is important to me that I uphold my commitments to significant people in my life*,” (4) “*Caring deeply about another person such as a close friend or relative is important to me*,” and (5) “*Knowing that a close other acknowledges and values the role that I play in their life makes me feel like a worthwhile person*.” I obtained a Cronbach’s alpha of 0.90 and averaged the items to form an index.

#### Relational identification

Relational identification was reported by supervisors in time 2 with the four-item relational identification scale developed by Sluss et al. (2012). To match the focus of this paper, the wording of the items was modified to assess the extent to which a supervisor identifies with the work relationship held with a specific subordinate. The resulting items are: (1) “*My work relationship with this subordinate is an important part of who I am at work*,” (2) “*If someone criticized my work relationship with this subordinate, it would feel like a personal insult*,” (3) “*My work relationship with this subordinate is vital to the kind of person I am at work*,” and (4) “*My work relationship with this subordinate is important to my self-image at work*.” I obtained a Cronbach’s alpha of 0.92 and averaged the items to form an index.

#### Family supportive supervisor behaviors

Family-supportive supervisor behaviors (FSSB) were reported by subordinates in time 2 using the shortened version of the FSSB scale. The FSSB short form, comprised of four items, was validated as a parsimonious and valid way to measure the superordinate FSSB construct (Hammer et al., 2013). Accordingly, subordinates rated the following items in relation to their direct supervisor: (1) “*Your supervisor makes you feel comfortable talking to him/her about your conflicts between work and non-work*,” (2) “*Your supervisor demonstrates effective behaviors in how to juggle work and non-work issues*,” (3) “*Your supervisor works effectively with you to creatively solve conflicts between work and non-work*,” and (4) “*Your supervisor organizes your work to jointly benefit the department or unit and yourself*.” I obtained a Cronbach’s alpha of 0.91 and averaged the items to form an index.

#### Control variables

To account for factors intervening at the supervisor-subordinate dyadic level, I controlled for the time the supervisor and subordinate had worked together in that hierarchical relationship, which was self-reported by the supervisor (measured in years), because relationship duration may influence how relationships unfold in work settings. In addition, I controlled for the supervisor’s gender (1 = female, 0 = male), the subordinate’s gender (1 = female, 0 = male), and the frequency of interaction with the target subordinate within the workplace. Supervisors self-reported the frequency of interaction in time 1 (1 = Never, 2 = Once per month, 3 = 2 or 3 times per month, 4 = 1 or 2 times per week, 5 = 3 or 4 times per week, 6 = 1 or 2 times per day, 7 = 3 + times per day).

### Analytical procedure

Given the nested structure of the data, 205 supervisor-subordinate dyads nested in 84 supervisors, I computed intra-class correlation coefficients for the four substantive variables to assess the within- and between-group variance. The ICC (1) scores have values of 0.29, 0.45, 0.34, and 0.21 (likeability, competence, supervisors’ relational identification, and FSSB, respectively), so I use a random intercept in the analysis to account for the variance at the supervisor level (Bliese, 2000).

The bruceR package in R was used to test the hypothesized relationships (see Figure 1) in two interrelated steps. In the first step, I used Hayes and Rockwood (2020) PROCESS Model 3 for multilevel-mediation analysis. After including control variables and random intercept, this model was used to test the effect of supervisor’s likeability and competence perceptions of the subordinate on supervisor’s relational identification with the subordinate (Hypothesis 1: Model 1 in Table 1), that of supervisor’s relational identification with the subordinate on subordinate’s perception of the supervisor’s FSSB (Hypothesis 2: Model 2 in Table 1), and the mediating mechanism that connects supervisor’s perceptions about the subordinate and the subordinate’s perception of the supervisor’s family supportive behaviors (Hypothesis 3: Models 1 and 2 in Table 1). To test Hypothesis 3, I conduct a formal significance test for the indirect effects and compute Monte Carlo confidence intervals based on 5,000 simulations.

**Table 1:** Mixed-effects analysis with random-intercept.

	DV:RI	DV:FSSB	DV:RI	DV:FSSB
	**Effect:Direct**	**Effect:Direct**	**Effect:Conditional**	**Effect:Conditional**
	**Model 1**	**Model 2**	**Model 3**	**Model 4**
Intercept	0.02	2.60**	3.32*	3.38
*Controls*				
Relational tenure	−0.02	0.02	−0.01	0.02
Frequency of interaction	−0.03	−0.01	−0.05	−0.02
Gender of supervisor	0.17	0.09	0.18	0.09
Gender of subordinate	0.01	0.09	0.03	0.10
Relational self-concept (RSE)	0.09	−0.16	−0.75*	−0.36
*Direct effects*				
Likeability	0.28** (H1a)	0.34**	−0.80	−0.58
Competence	0.43*** (H1b)	−0.08	0.66	0.69
Relational identification (RI)		0.19* (H2)		0.17*
*Interaction effects*				
RSE × Likeability			0.26* (H4a)	0.21
RSE × Competence			−0.05 (H4b)	−0.17
*N*	205	205	205	205
Clusters (Supervisors)	84	84	84	84
Pseudo *R*^2^	0.59	0.26	0.62	0.26
Random intercept variance	0.26	0.14	0.27	0.14
Residual variance	0.34	0.63	0.31	0.63

FSSB, family supportive supervisor behaviors; RI: relational identification with the subordinate. Relational tenure was measured in years, Frequency of interaction (1 = Never, 2 = Once per month, 3 = 2 or 3 times per month, 4 = 1 or 2 times per week, 5 = 3 or 4 times per week, 6 = 1 or 2 times per day, 7 = 3 + times per day), Gender of supervisor (1 = female, 0 = male), and Gender of subordinate (1 = female, 0 = male). Unstandardized coefficients reported; *p < 0.05; **p < 0.01; ***p < 0.001.

In the second step, I used Hayes and Rockwood (2020) PROCESS Model 7 for multilevel-mediation analysis. Using this model, I tested the interaction effects described in H4 (Model 3 in Table 1) and proceeded to analyze the effects of this interactions on the rest of the model by estimating conditional indirect effects (Models 3 and 4 in Table 1). More specifically, based on 5,000 simulations I computed indirect effects coefficients for high, mean, and low levels of the moderator variable (Supervisor’s relational self-concept).

## Results

### Confirmatory factor analysis

The lavaan package in R for confirmatory factor analysis was used to assess the convergent validity of the four variables at the supervisor-subordinate relational level (i.e., supervisor’s likeability perception of the subordinate, supervisor’s competence perception of the subordinate, supervisor’s relational identification with the subordinate, and subordinate’s perception of the supervisor’s family supportive behaviors). Table 2 shows that the hypothesized four-factor model had the best fit (*χ*^2^(98) 221.40, CFI = 0.95, TLI = 0.94, RMSEA = 0.08, SRMR = 0.05), which was significantly better than other models. For instance, as shown in Table 2, the four-factor model fits the data significantly better than the three-factor model where likeability and competence items load onto a single factor, than the three-factor model where relational identification and family supportive supervisor behaviors load onto a single factor, and the two-factor model where likeability, competence, and relational identification items load onto the same factor. Statistical analysis based on fit indices and chi-square significance tests reveals that the four variables the four variables at the supervisor-subordinate relational level represent different constructs. Table 3 displays the means, standard deviations, correlations, and internal reliability values of our study variables.

**Table 2:** Confirmatory factor analysis of measurement model (*N* = 205).

Model	*χ* ^2^	*df*	Δ*χ*^2^ (Δ*df*) test	CFI	TLI	RMSEA	SRMR
Model 1	221.40	98	–	0.95	0.94	0.08	0.05
Model 2	466.45	101	*p* < 0.001	0.86	0.84	0.13	0.07
Model 3	759.27	101	*p* < 0.001	0.76	0.71	0.18	0.16
Model 4	1040.48	103	*p* < 0.001	0.65	0.59	0.21	0.13
Model 5	1561.81	104	*p* < 0.001	0.46	0.37	0.26	0.19

All comparisons are relative to Model 1. Model 1: Four factor model. Model 2: Three factor model–likeability and competence items load onto the same factor. Model 3: Three factor model–relational identification and family supportive supervisor behaviors items load onto the same factor. Model 4: Two factor model–likeability, competence, and relational identification items load onto the same factor. Model 5: All items load onto a single factor. CFI, comparative fit index; TLI, Tucker-Lewis index; RMSEA, root mean square error of approximation; SRMR, standardized root mean residual.

**Table 3:** Means, standard deviations, correlations, and reliabilities (*N* = 205).

		***M***	**SD**	**1**	**2**	**3**	**4**	**5**	**6**	**7**	**8**	**9**
1	Relational tenure	3.26	1.83	–								
2	Frequency of interaction	6.80	0.67	0.01	–							
3	Gender of supervisor	0.54	0.50	−0.04	−0.08	–						
4	Gender of subordinate	0.38	0.49	0.01	−0.08	0.29***	–					
5	Relational self-concept	4.39	0.61	−0.01	−0.06	0.07	−0.00	**0.90**				
6	Likeability	4.13	0.66	−0.07	0.01	−0.02	−0.09	0.30***	**0.90**			
7	Competence	4.01	0.67	−0.11	−0.04	0.11	−0.08	0.34***	0.66***	**0.92**		
8	Relational identification	3.06	0.86	−0.12	−0.06	0.11	−0.1	0.25***	0.38***	0.44***	**0.92**	
9	Family supportive supervisor behaviors	3.60	0.91	−0.01	−0.02	0.07	0.07	−0.02	0.26***	0.15*	0.20**	**0.91**

Relational tenure was measured in years, Frequency of interaction (1 = Never, 2 = Once per month, 3 = 2 or 3 times per month, 4 = 1 or 2 times per week, 5 = 3 or 4 times per week, 6 = 1 or 2 times per day, 7 = 3 + times per day), Gender of supervisor (1 = female, 0 = male), and Gender of subordinate (1 = female, 0 = male). Reliabilities in bold in diagonal.

*p < 0.05; **p < 0.01; ***p < 0.001.

### Descriptive statistics analysis

As reported in Table 3, the bivariate correlations lend initial support for the hypothesized relationships about direct effects. More specifically, impressions reported by supervisors in time 1 about subordinate’s likeability (H1a: *r* = 0.38, *p* < 0.001) and competence (H1a: *r* = 0.44, *p* < 0.001) are positively related to relational identification with subordinates at time 2. Also, relational identification with subordinates is positively related with FSSB (H2: *r* = 0.20, *p* < 0.01), as reported by subordinates in time 2. Also, none of the control variables showed a systematic association with the key constructs in this study.

### Hypothesis testing

When relational tenure, frequency of interaction, gender of supervisor, gender of subordinate, and relational self-concept of supervisor were controlled (see Table 1), the supervisor’s likeability perception of the subordinate was positively related to the supervisor’s relational identification with the subordinate (Model 1: *B* = 0.28, *p* < 0.01), supporting Hypothesis 1a. Similarly, the supervisor’s competence perception of the subordinate was positively related to the supervisor’s relational identification with the subordinate (Model 1: *B* = 0.43, *p* < 0.001), supporting Hypothesis 1b. Moreover, after including supervisor’s likeability and competence perceptions of the subordinate as additional controls, the supervisor’s relational identification with the subordinate relates positively to FSSB (Model 2: *B* = 0.19, *p* < 0.05); thus, Hypothesis 2 was supported.

H3 predicts that supervisor’s perceptions about the subordinate on (a) likeability and (b) competence have an indirect effect on FSSB *via* supervisor’s relational identification. As derived from the empirical tests for H1a (Model 1: *B* = 0.28, *p* < 0.01) and H2 (Model 2: *B* = 0.19, *p* < 0.05), the coefficient for the indirect effect of likeability perceptions on FSSB *via* relational identification is 0.05, and the 95% CI [0.00, 0.12] does not zero, indicating that the indirect effect hypothesized in H3a is significant. In a similar vein, the indirect effect coefficient for testing H3b is 0.08, and the 95% CI [0.01, 0.17] does not include zero, lending support for the indirect effect of competence perceptions on FSSB *via* relational identification. In sum, H1, H2, and H3 were fully supported.

Hypothesis 4 predicts that supervisor’s relational self-concept will moderate the relationship between the supervisor’s likeability (H4a) and competence (H4b) perceptions and supervisor’s relational identification with the subordinate, such that the relationships would be stronger for those cases where supervisors rate high on relational self-concept. As shown in Model 3 of Table 1, H4a was supported–the interaction between supervisor’s likeability perception and supervisor’s relational self-concept is significantly related to supervisor’s relational identification with subordinate (Model 3: *B* = 0.26, *p* < 0.05). However, H4b was not supported–the interaction between supervisor’s competence perception and supervisor’s relational self-concept is not significantly related to supervisor’s relational identification with subordinate (Model 3: *B* = −0.05, *p* > 0.1).

**Figure 2:**
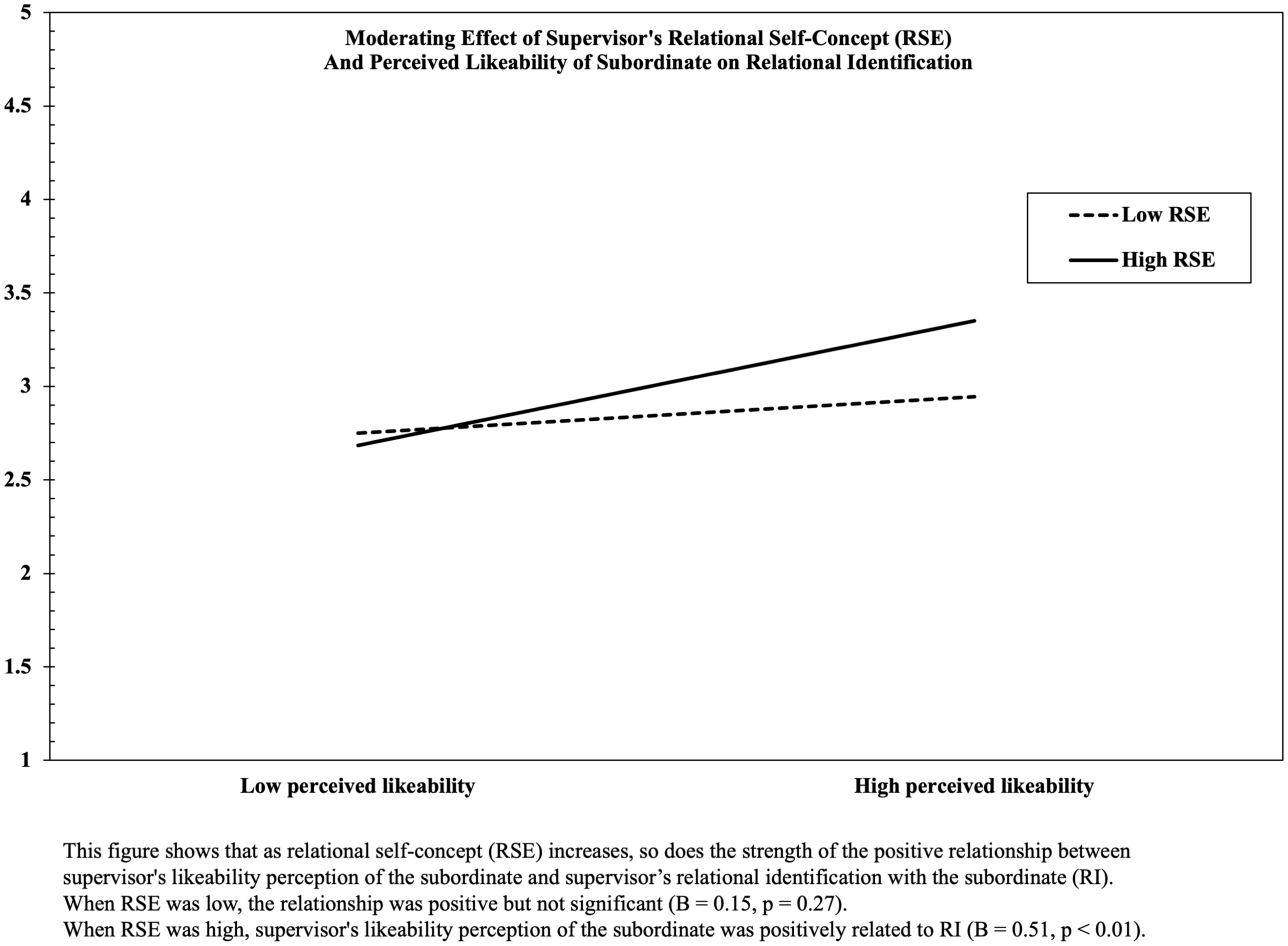
Moderating effect of supervisor’s relational self-concept (RSE) and perceived likeability of subordinate on relational identification (RI).

Simple slope analysis was performed to further demonstrate H4a, the interaction effect between supervisor’s likeability perception and relational self-concept. Simple slope analysis, as shown in Figure 2, revealed that the effect of supervisor’s likeability perception on relational identification with subordinate was stronger when relational self-concept was high (*B* = 0.51, *p* < 0.01) than when low (*B* = 0.15, *p* = >0.1). To further verify the conditional indirect effect, I computed indirect effect coefficients at high and low levels of the moderator variable. In line with the simple slope analysis, the 95% CIs indicate that the indirect effect of likeability perceptions on FSSB *via* relational identification was stronger when relational self-concept was high [0.00, 0.19] than when low [−0.02, 0.09]. In other words, the indirect effect of perceived likeability gets stronger as relational self-concept increases.

## Discussion

The objective of this paper was to study the antecedents of family supportive supervisor behaviors (FSSB) from a relational perspective. As hypothesized, the results of the present study supported the expected relationship between relational identification with subordinates and FSSB. Supervisors who incorporated the work relationship with the subordinate to their definition of self were perceived as family supportive by the subordinate. Moreover, supervisor’s impressions about the likeability and competence of a target subordinate positively impact their relational identification with the subordinate. Yet, the positive effect of perceived likeability on relational identification is weaker for supervisors who rate low on relational self-concept.

### Theoretical implications

One of the purposes of this article was to better understand the nomological network of FSSB. In line with previous research (e.g., Hammer et al., 2007; Straub, 2012; Sargent et al., 2022), here I frame FSSB as a discretionary behavior. Moreover, in the present study I show that the extent to which a supervisor identifies with the work relationship he or she holds with a subordinate is positively related to the actions they take to help the subordinate address and manage their work and personal life responsibilities. The identity lens adds to the already existing literature on supervisory discretionary behavior to better understand why supervisors engage in behaviors that benefit others. The type of behaviors here are neither related to specific work tasks nor to interpersonal problems that arise within the workplace. Supervisors who engage in FSSB demonstrate understanding and support for the difficulties that employees face at work and provide assistance for problems that employees face in their personal lives (Crain and Stevens, 2018). Therefore, the underlying exchange-based logic present in current theories, where supervisors help subordinates with the expectation of receiving something positive in return from them, may not hold for studying behaviors where the instrumentality (i.e., organizational and economic benefits that would accrue from helping) for the provider of help is uncertain (Toegel et al., 2013).

Work-family scholar have advocated for the use of exchange-based theories in order to better understand the antecedents and consequences of FSSB (Matthews and Toumbeva, 2015). The perspective I take here incorporates the exchange-based logic because the relationships between supervisors and subordinates are defined in the basis of a work contract. However, it expands this logic by focusing on a type of liaison that develops through work interactions that has the potential to become a component of how supervisors define themselves at work (Sluss and Ashforth, 2007). The results of the present study suggest that moving beyond exchange-based theories may be necessary for the type of behaviors studied here. For example, in the present study I found that the effect of likeability on FSSB is partially mediated by relational identification and that the effect of competence is fully mediated. The full mediation reported for competence may suggest that the decision to engage in FSSB by supervisors is not grounded on the direct gains they may obtain by helping competent subordinates (e.g., reputation with their managers, being more effective as supervisors themselves). Thus, I contribute to the literature on supervisor discretionary behaviors and specifically to the work-life literature by showing that supervisors engage in behaviors that help subordinates because they ultimately feel that they are helping themselves too in ways that are not as calculative as exchange-based theories propose.

A related purpose of this research was to contribute to the relational identity literature by better understanding the antecedents of relational identification with subordinates. First, I found that subordinates who are perceived as likeable engender a higher degree of relational identification in their supervisors. Second, I also found that supervisors develop a higher degree of relational identification with those subordinates they perceive as competent. Moreover, in this study perceived likeability and competence had a positive indirect effect through relational identification with subordinates. This mechanism is an important contribution to the management literature because although previous scholars have acknowledged that perceptions of likeability and competence affect the performance appraisal done by supervisors (e.g., Diekmann et al., 2015), this is the first study showing that these effects may occur because those perceptions engender feelings where the target and the self tend to overlap. Moreover, the pervasiveness of the overlap is such that, as studied here in the context of supportive behaviors, it translates to behaviors that do not directly relate to the task-related goals and objectives of the organization–FSSB.

Regarding the boundary conditions underlying the relationship between impressions about subordinates and relational identification with subordinates, not all the hypothesized relationships were supported. Building on theories of self-concept (e.g., Cross et al., 2000; Johnson et al., 2006), I hypothesized that for supervisors with a higher relational self-concept the effect of likeability and competence impressions on relational identification would be stronger. These hypotheses were developed based on the assumption that both dimensions of interpersonal impressions — likeability and competence — are equally important to the perceiver in the workplace because they convey the valence of the target for establishing and maintaining meaningful work relationships. Nevertheless, the results of the moderation analysis revealed that although both dimensions of interpersonal impressions have a substantial effect on relational identification, the impact of likeability perceptions differed from that of competence perceptions. Specifically, relational self-concept qualified the relationship between perceptions of likeability and relational identification, but not the relationship between perceptions of competence and relational identification.

A possible explanation for the mixed support for this hypothesis is that relational self-concept is conceptually closer to likeability than to competence. Whereas competence relates to tasks and capabilities to be effective, likeability relates to intentions and trustworthiness in interactions. Therefore, likeability is conceptually closer because it captures the characteristics that meet the relational needs strived by individuals with a salient relational self-concept. Research by Frame et al. (2010) has reported that supervisors, irrespective of their gender, value in others competence-related attributes more than likeability-related attributes within the workplace. Moreover, their findings also convey that the value supervisors give to likeability-related traits may have a greater variance, since female supervisors (argued to have a higher relational self-concept than their male counterparts) value more these traits in others than male supervisors. It can be the case, then, that characteristics of others related to building relationships are relevant for the process of identification only for those individuals that are more attracted to those aspects at the trait level. In a related manner, if competence is less directly related to relational self-concept than likeability, the non-significant interaction may have been due to the resulting high mean for relational self-concept and the possibility of a resulting ceiling effect where the lack of significance may be due to the low level of variability found in supervisors’ reports of relational self-concept. In fact, previous field research has reported already that perceptions about others’ competence has a much lower variability than perceptions of others’ likeability (Casciaro and Lobo, 2008).

### Practical implications

An important caveat that organizations need to be aware of is that supervisors may fail to provide support to those who need it because their level of relational identification is low. In those cases, organizations may fail to obtain the positive outcomes that are associated with FSSB [i.e., employee creativity (Zhou et al., 2022), task performance (Wang et al., 2013), employee perceived health (Rofcanin et al., 2020), etc.], resulting in organizational outcomes that are far from ideal. Therefore, organizations may want to consider ways by which the discretion of supervisors in deciding whether to engage in FSSB with all subordinates decreases. For example, organizations might consider adding FSSB to job descriptions of supervisors and including it as part of their performance appraisal process. They might also think of ways to help remind supervisors about connecting with subordinates as a medium by which they learn about the current struggles their subordinates face when integrating work and non-work demands. For example, Hammer et al. (2011) conducted an intervention to develop the supervisors’ competencies for providing family supportive supervisor behaviors. Training sessions communicated that the organization encouraged those behaviors and developed the competencies on empathy and corner for others’ difficulties integrating work and life demands. This training resulted in increased supervisor knowledge about FSSB, produced increases in self-set goals for delivering those behaviors, and also in increases in the delivery of those behaviors (Hammer et al., 2011). Thus, training sessions where this kind of behavior is framed as part of their responsibilities as supervisors and where the required skills are developed may help limit favoritism when showing concern and empathy for others’ work-life integration struggles.

In fact, the two antecedents studied here convey that relational identification neither occurs in a vacuum nor is constant. These antecedents offer a novel lens for better understanding subordinates as more than mere recipients of supervisory behaviors. Other scholars in the work-life literature have conceptualized FSSB as the discretionary behavior associated to the identification with specific social categories present in the workplace (e.g., Foley et al., 2006; Li and Bagger, 2011). The results of the present study suggest that the implications of those beliefs may be misleading if the actual perceptions of supervisors are not taken into account, since the recipients of support may actively engage in tactics that will help them build the desired images and thereby develop a more supportive relationship with their supervisor (Turnley and Bolino, 2001). For example, with the objective of building better relationships, organizations can try to attract employees who have greater interpersonal abilities to convey their likeability and competence to supervisors. For example, Turnley and Bolino (2001) show that self-monitors are better able to build desired images while avoiding undesired ones. Similarly, Diekmann et al. (2015) report that trait modesty of subordinates, coupled with modesty impression management tactics, positively relates to likeability and competence perceptions done by supervisors. However, organizations should warn supervisors about the negative consequences they may face if they decide to be supportive based on interpersonal impressions. By helping people they like, they overlook the needs of the most vulnerable employees–those not capable of impression management.

### Limitations

Several limitations help specify the actual scope of the theoretical and practical contributions of our research. First, limitations to the external validity of our results come from a sample Chilean supervisors and subordinates from a private bank. In different organizational and cultural contexts, different type of behaviors may be needed to better integrate work and non-work demands (Powell et al., 2009). Therefore, the extent to which findings can be generalized to other contexts should be carefully considered. Accordingly, more research in other organizational and cultural settings is required to test the proposed relationships and develop more robust recommendations for supervisors.

Second, the interpretation of findings is constrained by the measure of FSSB used in the present study. This measure was the first to be developed to evaluate such behaviors; yet, it is not exempt from construct validity issues. In fact, although the scale used in the present study is comprehensive, it does not capture the complete range of behaviors that are needed by employees to balance work and family (see: Hammer et al., 2009, 2013). Thus, the present study is not able to rule out the effect of other supervisor behaviors that may help employees better integrate work and non-work responsibilities in this or other alternative settings (i.e., virtual work). Future research should focus on developing scales that capture a broader range of family supportive behaviors as stronger results could probably emerge from a more comprehensive measure.

Third, the multisource nature of the dyadic data is among the strengths of this study. The use of data from both superiors and subordinates reduces the likelihood that the relationships discovered in the study are due to common source bias. T2 variables were measured 4 weeks after T1 variables, but the data are nonetheless cross-sectional. Thus, I am unable to establish causal relationships between the constructs of this study. Future research may employ longitudinal research designs to investigate the causal nature of the relationships I’ve proposed here in greater depth. Also, in relation to the data collection procedures, I did not account for missing data. Due to an earthquake, data collection for time 1 variables was halted 1 day ahead of schedule. This earthquake kept respondents away from the office, resulting in incomplete supervisor responses. As a result, a number of subordinates were not rated for likeability and competence, and supervisors were unable to self-report their relational self-concept; consequently, they were excluded from the analysis. It is well documented in the missing data literature that by dropping those cases there is a loss of power in the statistical analysis (Newman, 2014), which may be reflected in the size of the effects reported. It may well be the case that the loss of power explains the lack of significance found in the self-concept competence interaction. Therefore, further developments on how to deal with missing data when data has a nested structure would be beneficial to conduct a more rigorous analysis.

### Conclusion

In the present article, I examined the antecedents of family supportive supervisor behaviors (FSSB) from a relational perspective. I found that within work-units, subordinates do not perceive supervisors equally in terms of FSSB and that the associated variance is explained by the level of relational identification with subordinates as reported by supervisors. Additionally, supervisors develop relational identification with subordinates based on their impressions about subordinates in two dimensions: competence and likeability. I hope that the present findings encourage work-life scholars to further explore the antecedents of FSSB from a relational perspective.

## Data availability statement

The datasets presented in this article are not readily available because it is part of a larger research program that is currently in progress. Requests to access the datasets should be directed to PE, p.escribano@uai.cl.

## Ethics statement

Ethical review and approval was not required for the study on human participants in accordance with the local legislation and institutional requirements. The patients/participants provided their written informed consent to participate in this study.

## Author contributions

The author listed has made a substantial, direct, and intellectual contribution to the work, and approves it for publication.

## Conflict of interest

The author declares that the research was conducted in the absence of any commercial or financial relationships that could be construed as a potential conflict of interest.

## Publisher’s note

All claims expressed in this article are solely those of the authors and do not necessarily represent those of their affiliated organizations, or those of the publisher, the editors and the reviewers. Any product that may be evaluated in this article, or claim that may be made by its manufacturer, is not guaranteed or endorsed by the publisher.
